# Winter wheat phenotyping for deep root growth and function, reduced water stress and increased uptake of deep N and water

**DOI:** 10.1093/aob/mcaf160

**Published:** 2025-08-01

**Authors:** Arnesta Odone, Satyasaran Changdar, Kristian Thorup-Kristensen

**Affiliations:** Department of Plant and Environmental Sciences, University of Copenhagen, Taastrup 2630, Denmark; Department of Computer Science, University of Copenhagen, Copenhagen Ø 2100, Denmark; Department of Plant and Environmental Sciences, University of Copenhagen, Taastrup 2630, Denmark

**Keywords:** Root physiology, deep roots, *Triticum aestivum*, water stress, isotopic tracer, ^15^N, ^2^H, ^13^C, carbon isotope discrimination

## Abstract

**Background and Aims:**

Deep roots may help plants adapt to climate change by allowing them to access deeper soil layers where water is still available, reducing water stress and increasing nitrogen (N) uptake. Water stress significantly affects yield during later developmental stages, but methods are lacking for phenotyping for deep rooting under field conditions and at maturity.

**Methods:**

Over 3 years, we used minirhizotron root imaging in the RadiMax semi-field facility to compare deep rooting in winter wheat genotypes grown in field soil to 2.7 m depth. We related this to deep soil uptake of water and N using isotopic tracers injected into the soil at 1.6–1.8 m depth. Carbon isotope discrimination was used to evaluate water stress levels.

**Key Results:**

Deep rooting was positively correlated with uptake of deep-placed N and water, and uptake of deep-placed N was three times higher in the genotype with deepest roots compared with the shallowest. Deep rooting was negatively correlated with water stress, measured using carbon isotope discrimination. This correlation was strongest in 2023, a dry year, highlighting the role of deep roots in mitigating water stress. Some genotypes had consistently deeper or shallower roots over the three experimental years, and there were strong correlations of isotopic measurements between genotypes across years.

**Conclusions:**

Our findings show strong relationships between deep rooting and deep root functions, which indicate that deep rooting is a desirable trait that should be targeted. The significant genotypic variation observed, which can be phenotyped for even under field conditions, indicates that deep rooting is a trait that can be incorporated into breeding programmes. Furthermore, the methods used in this study are effective and should be developed for further application.

## INTRODUCTION

Climate change is increasingly causing extreme weather events which are affecting crop production globally. In Northern Europe, climate change is generating wetter winters and drier summers (IPCC, 2023). This can lead to substantial leaching of mobile nutrients in winter (Ulén and Johansson, 2009; Øygarden *et al*., 2014), while increased water stress in spring and summer can significantly reduce yields (Sionit *et al*., 1980; Wang *et al*., 2017). More resilient crops are needed that can maintain yields in this increasingly extreme climate.

One potential solution is to grow deeper-rooting crops. Under water-stressed conditions when the upper soil layers are dry, deep roots can access soil layers where water is still available, particularly in the later stages of crop development (Passioura, 1983). Additionally, when there is high precipitation, mobile nutrients such as nitrate are leached into deeper soil layers. Crops with deeper roots can take up deep nitrate (Kristensen and Thorup-Kristensen, 2004; Wacker *et al*., 2022; Odone *et al*., 2023), reducing leaching losses to the environment. However, despite their importance, root traits have been generally neglected in plant breeding due to the difficulty of accessing and assessing them.

To address this, methods are needed for phenotyping roots, especially deep roots. Methods in pots and greenhouses are inadequate for evaluating the full mature root system, and have not been shown to relate to root development in the field (Bai *et al*., 2019; Rich *et al*., 2020). To evaluate roots to their full capacity, including the importance of deep roots, crops should be grown to full development in a realistic deep soil profile. In the field, previous studies have already demonstrated variation in deep rooting in wheat (Botwright Acuña and Wade, 2012; Rasmussen *et al*., 2015; Odone and Thorup-Kristensen, 2025*b*). However, on a scale suitable for phenotyping there are very few examples. Root phenotyping therefore needs to be tested on a larger scale, better quantified and related to deep resource use. Additionally, there is no standardized measure of what constitutes a deep root (Maeght *et al*., 2013); different root traits or architectures should be assessed for their contribution to water and nutrient uptake, and to determine their value under different conditions.

Developing methods to allow large-scale phenotyping of roots will enable breeders to measure what is otherwise ‘the hidden half’ and incorporate roots into future crop breeding. By including deep rooting as a target trait in breeding programmes, plant breeders can shift their focus from maximizing yield potential to also ensuring yield stability, with crops that can continue to produce under a range of different conditions. This would mean selecting for specific traits that are advantageous under adverse climate conditions, rather than solely for high yields.

In this study we present results from 3 years of experiments in the RadiMax facility (Svane *et al*., 2019) evaluating deep root development in winter wheat. The facility is designed to phenotype for deep roots; by imaging the root systems we can evaluate their size and development in real soil, and use isotopic tracer methods to test how much water and nitrogen (N) they take up from depth. This research is important for identifying whether there are differences in deep rooting between genotypes, and determining if these differences help crops to exploit the subsoil and withstand the stresses of a changing climate.

Therefore, we pose the following research questions: (1) Are there genotypic differences in deep rooting and can we phenotype for these differences? (2) Do genotypes with deep roots have a higher uptake of deep soil water and N?

## MATERIALS AND METHODS

### The RadiMax root phenotyping facility

**Fig. 1.**
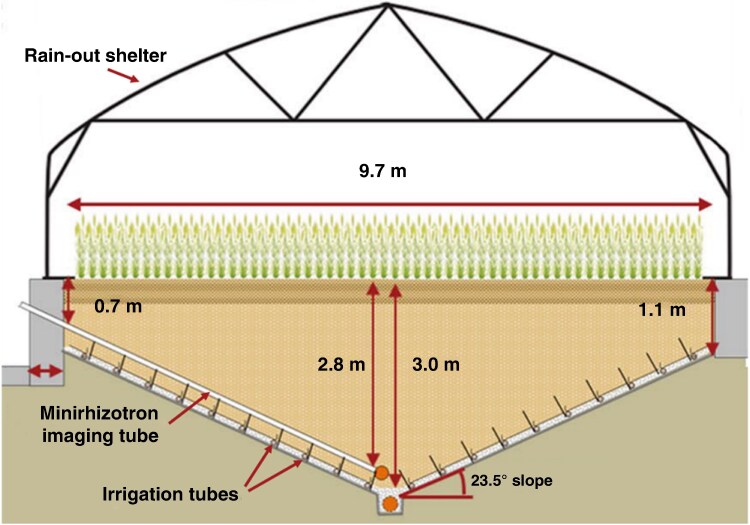
A cross-section of the RadiMax facility, showing minirhizotron imaging tubes, rain-out shelter and irrigation tubes. Adapted from Svane *et al*. (2019).

Winter wheat was grown in the unique RadiMax root phenotyping facility (Svane *et al*., 2019) over 3 years, 2021–23. The RadiMax facility is located in Taastrup, Denmark (55.66815°N, 12.30848°E). The facility consists of four 9.7 m × 40 m long concrete beds, which have a V-shaped base (Fig. 1). In the experiments from 2021–23, the deeper two beds of the facility were used, with soil reaching 3 m depth in the centre of the beds, and shallower soil at the edges. Crops were grown in 150 single rows per bed, 9.7 m long, with rows spaced 0.25 m apart. A transparent acrylic minirhizotron tube is permanently placed below each crop row, descending along the V-shaped base, from the shallow edge of the bed to the deepest part in the middle of the bed. The tubes are 5.5 m long, with a 70 mm external diameter, and placed at a 24° angle sloping downwards, allowing root observation from 0.6 m depth at the edge to 2.7 m at the centre of the bed.

Water sensors (TDT volumetric water content (VWC) (Acclima, Inc., Boise, ID, USA)) for soil water monitoring are placed along the slope of each bed at 0.5-m intervals (0.2 m soil depth), at two positions within each bed, and centrally within the beds at two locations at 0.5-m depth intervals. Irrigation tubes run lengthwise along the base of the beds at 0.5-m intervals (0.2 m soil depth), which allow subsoil injection of isotopic tracers and irrigation at different depths. The soil in the facility was filled in 2015 with homogenized subsoil from a nearby area (sandy loam in the Danish soil classification), topped up with 0.4 m of topsoil. Further details about the construction of the RadiMax facility are described by Svane *et al*. (2019).

### Winter wheat cultivars and experimental design

In 2021, nine modern Danish winter wheat cultivars were replicated in at least six crop rows. Additional rows were used for single-row phenotyping but excluded in the present analysis. In 2022 and 2023, these same nine cultivars were replicated ten times in each year, and an additional 39 genotypes were replicated in four crop rows. These extra genotypes were a mixture of modern breeder lines and cultivars. The experiment was arranged in a randomized complete block design, within two beds of the RadiMax facility. Rows of LG Skyscraper were replicated every seven to ten rows as a control for variation within the beds, both for root and shoot development. Cultivar information is in Supplementary Data Table S1.

### Management

Winter wheat was sown each year in early October, following a cover crop mixture of yellow mustard, oilseed radish and phacelia, which were sown directly after harvesting the previous wheat crop. The wheat was sown in individual rows 9.7 m long, with 500 seeds per 10-m row, and 0.25 m between rows. They were fertilized according to local recommendations. Rain-out shelters were placed over the facility post-anthesis in 2021 and 2022 to induce drought during grain filling. These were not used in 2023, as drought occurred naturally. See Table 1 for dates.

**Table 1.** Management, isotope application and imaging dates of winter wheat experiments in RadiMax 2021–2023.

	2021	2022	2023
Sowing	6–7 October 2020	6–7 October 2021	5–6 October 2022
Fertilization 1	18 March	21 March	29 March
*(kg N ha^−1^)*	90	91	87
Fertilization 2	29 April	25 April	1 May
*(kg N ha^−1^)*	95	75	77
Imaging 1	11–12 May	17–18 May	–
Imaging 2	7–9 June	7–9 June	6–8 June
Imaging 3	6–8 July	13–14 July	11–13 July
Rain-out tents on	21 June	20 June	–
Isotope injection	15 June	8 June	7 June
Harvest	27–28 July	26–27 July	18–19 July

### Isotopic tracer application

Isotopic tracers of ^15^N (Ca^15^NO_3_) and ^2^H-labelled water (^2^H_2_O) were injected into subsoil irrigation lines running along the base of each side of the beds at 1.6 and 1.8 m soil depth. The isotope mixture of 54.6 L water, 1.51725 g ^15^N (8.925 g CaNO_3_) and 1 L ^2^H-labelled water were injected into irrigation lines running under both the north and south side of each bed, giving the equivalent of 0.00759 g ^15^N and 5 mL ^2^H-labelled water to the north and south side of each crop row. Isotope injection was timed at mid-anthesis; see Table 1 for dates.

### Plant sampling

Plants from a 0.5-m long section of crop row directly above the isotope injection were harvested at maturity. Grain samples were taken for all 300 crop rows in the experiment, and straw was also sampled in 30 rows in each bed. Additionally, plant samples were taken from an area above the shallow and deep soil areas within each row at maturity, to compare grain yield and biomass. All samples were dried at 80 °C for 24 h and threshed. Grain and straw samples were weighed, and then finely milled and analysed for ^13^C and ^15^N at the Stable Isotope Facility, IGN, Copenhagen University, and for ^2^H at the Centre for Stable Isotope Research and Analysis, University of Göttingen.

### Root imaging and image analysis

Images of the roots were taken using a semi-automated minirhizotron camera imaging system, which takes images every 35 mm of the upper side of the tube (Videometer, Herlev, Denmark). Imaging began at 0.6 m soil depth, below the concrete edge of the facility, and taken down to 5500 mm tube depth (3 m soil depth). Images were taken at three time points during each growing season in 2020–21 and 2021–22, and twice in 2022–23. See Table 1 for imaging dates. RootPainter software (Smith *et al*., 2022) was used to detect root length in each image in pixels; these data were then converted to root length in centimetres for the soil depth interval of each image, and used to create the root profiles, and then to calculate a number of root traits for each crop row. These traits include DeepRoot_40_, which is the soil depth below which 40 cm of cumulative roots are observed within each minirhizotron tube. This has been defined and tested in the RadiMax facility as a measure of deep rooting correlating to deep soil N uptake (Wacker *et al*., 2022; Odone *et al*., 2023). Further root traits including root lengths in different soil intervals and sigmoid inflection (SI) (Changdar *et al*., 2023) were also compared with isotope uptake. Sigmoid inflection as an estimate of rooting depth was estimated by fitting a sigmoid function to the root length data along the minirhizotrons, and estimates the point where root density shifts from higher values in the upper part of the minirhizotrons to low values at the deeper part; this inflection point is used as a proxy for rooting depth.

### Data analysis

Data were analysed using R version 4.3.1. Data were analysed using generalized least squares (gls) models using the nlme package in R (Pinheiro, 2024). ANOVA was performed to assess the significance of cultivar, year and deep rooting (independent variables) on grain yield, deep rooting and isotope measurements (dependent variables). Row/bed/year were included within a correlation structure to account for spatial variation within the RadiMax facility. Log transformation was used for ^15^N and ^2^H models, with ^2^H + 90 used to account for negative values. Pairwise comparisons were conducted on the estimated marginal means of cultivars and years, using the emmeans package (Lenth, 2022). The nine main cultivars, which had four or more replications in each of the three years, are included in statistical models.

Correlation matrices of root lengths in different soil depth intervals were used to identify soil depths important for tracer uptake, and to identify relevant deep root traits. All correlations were conducted using Pearson correlations, using cultivar means within each experiment. All 48 cultivars with four or more replications within the experimental year are included in correlations.

## RESULTS

### Weather and water use

**Fig. 2.**
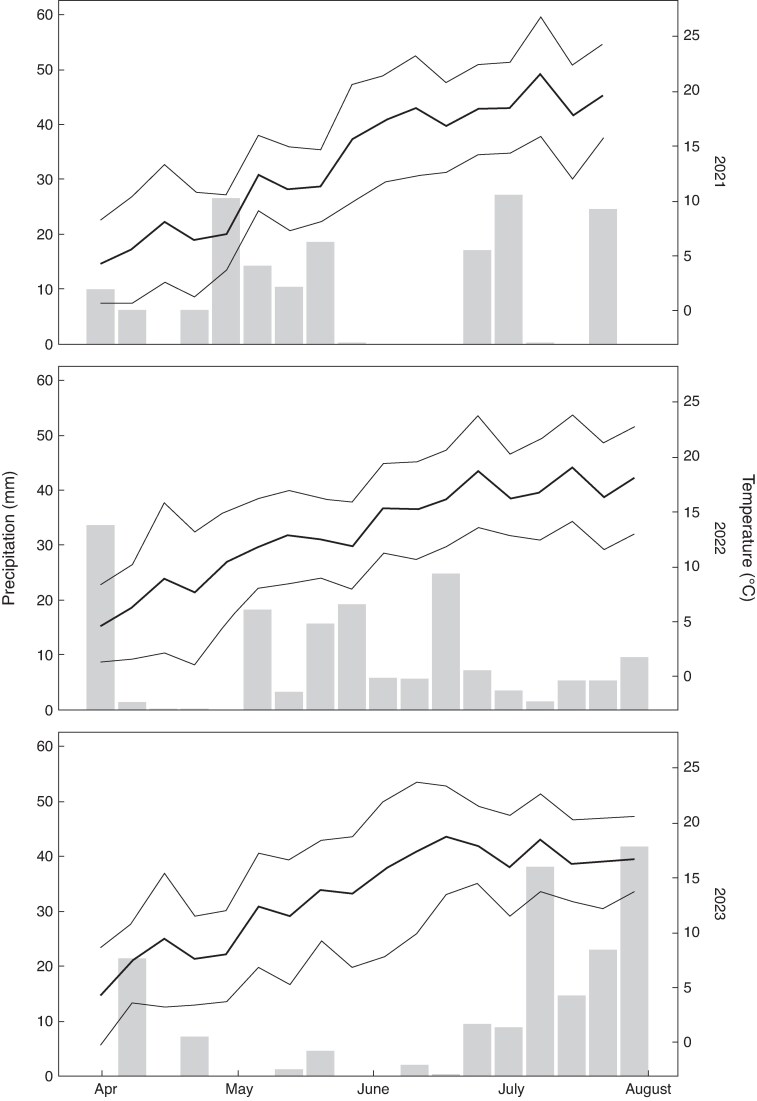
Weekly total precipitation (bars) and average weekly minimum, mean and maximum temperatures (lines) from April to August in each experimental year. Note that in 2021 and 2022 rain-out tents were used from mid-June. Data from Taastrup climate station (Svane *et al*., 2024).

The weather patterns over the three years varied significantly (Fig. 2). In 2021 the temperature and rainfall were average for the region, with a dry winter followed by a wet spring and a hot dry summer. 2022 was a warm year, with a dry spring following a mild and wet winter. In the early summer of 2023 there was very little precipitation, and high temperatures until the end of June.

**Fig. 3.**
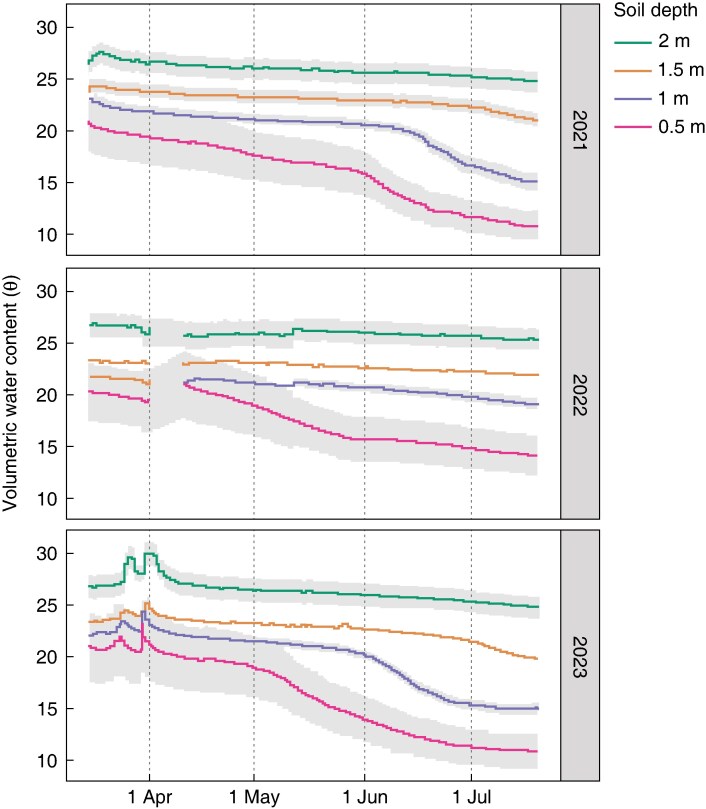
Soil volumetric water content measured in the RadiMax facility at 0.5, 1, 1.5 and 2 m soil depth. Grey bars show the standard error for water sensors at the same soil depth.

The soil VWC in RadiMax shown in Fig. 3 demonstrates much less water use in 2022 than in the two other years. The fastest and strongest depletion of soil water occurred in 2023, with a rapid reduction in water at 0.5 m from May, and at 1 m from early June, while in 2021 the water content reduced slowly from April at 0.5 m depth, and at 1 m from mid-June. In both years, significant water use was evident from the top 1 m depth and some from 1.5 m, with a slight indication of water use from 2 m at the end of the season.

### Yield, root development and isotope uptake

Grain yield was significantly lower in 2023 than in the previous years (Table 2). In 2021 and 2022, grain yield was slightly higher in the shallow sample than the deep sample, while in 2023 the grain yield was substantially higher in the deep sample. DeepRoot_40_ was higher in 2021 than 2022, and intermediate and not significantly different in 2023 than the other years. There was a significant cultivar effect on DeepRoot_40_ (see Table 4 for *P*-values).

**Table 2.** Yield overview for each experimental year in RadiMax. Grain yields for shallow and deep areas are from 60 selected crop rows, for eight cultivars. Total yield and root measurements are for all lines. Generalized least squares models were used for grain yield and DeepRoot40. Letters indicate significant differences between years (*P* < 0.05).

Year	Grain yield (g m^2^)						DeepRoot_40_ (cm)	
	Total		Shallow		Deep			
	Mean	s.e.	Mean	s.e.	Mean	s.e.	Mean	s.e.
2021	608*a*	7	629*a*	7	587*a*	8	116.2*a*	1.9
2022	606*a*	15	664*a*	17	548*a*	17	94.2*b*	1.0
2023	302*b*	11	191*b*	10	412*a*	14	111.1*ab*	1.5

There was uptake of labelled N (^15^N) and water (^2^H) tracer in all three years (Table 3). Grain δ^15^N values were significantly higher in 2023 than in the previous years, and lower in straw (Table 3). There were significant effects of cultivar, year, cultivar × year and DeepRoot_40_ on grain δ^15^N, and of cultivar, year and DeepRoot_40_ on straw δ^15^N (Table 4). There was uptake of ^2^H tracer in the grain in all three years, which was not significantly different between years. There was a significant effect of cultivar, year and DeepRoot_40_ on δ^2^H. Both grain and straw δ^13^C were significantly different between all three years, and were lowest in both grain and straw in 2023. There were significant effects of cultivar, year and cultivar × year on grain and straw δ^13^C, and of DeepRoot_40_ on straw δ^13^C.

**Table 3.** Isotope overview for each experimental year in RadiMax. Recovery of ^15^N is calculated using the total enrichment relative to the natural abundance of ^15^N, as a percentage of the total tracer applied divided by the 300 rows.

Year	δ^15^N				N in dry matter (%)				^15^N recovery (%)				δ^13^C				δ^2^H	
	Grain		Straw		Grain		Straw		Grain		Straw		Grain		Straw		Grain	
	Mean	s.e.	Mean	s.e.	mean	s.e.	Mean	s.e.	Mean	s.e.	Mean	s.e.	Mean	s.e.	Mean	s.e.	Mean	s.e.
2021	25.0	3.0	23.5	2.2	2.46	0.02	0.68	0.01	78.8	9.3	26.5	2.8	−26.5	0.05	−28.3	0.04	−48.6	1.3
2022	27.8	4.8	15.1	2.0	2.23	0.03	0.53	0.01	70.8	12.3	12.6	1.9	−25.7	0.04	−27.8	0.05	−55.4	0.3
2023	32.8	5.8	7.5	1.0	1.71	0.02	0.34	0.02	63.1	13.1	2.2	0.4	−24.8	0.04	−24.4	0.07	−51.2	1.4

**Table 4.** ANOVA table of variables affecting each of the indicators of deep rooting or deep root uptake, showing *F* and *P* values and degrees of freedom. Generalized least squares models were used with cultivar, year, DeepRoot40 and cultivar × year interactions as fixed effects and row/bed/year as a spatial correlation structure. Models for δ^15^N and δ^2^H were log-transformed. Cultivar d.f. = 9; year d.f. = 2.

	Grain yield		DeepRoot_40_		δ^13^C grain		δ^13^C straw		δ^15^N grain		δ^15^N straw		δ^2^H grain	
	*F*	*P*	*F*	*P*	*F*	*P*	*F*	*P*	*F*	*P*	*F*	*P*	*F*	*P*
Cultivar	9.6	<0.0001	6.9	<0.0001	21.34	<0.0001	25.3	<0.0001	4.19	0.0001	2.9	0.006	4.54	0.0001
Year	50.6	<0.0001	3.9	0.0229	29.05	<0.0001	365.7	<0.0001	3.19	0.0437	9.5	0.0002	1.02	0.363
DeepRoot_40_	7.3	0.0079			1.87	0.1727	7.9	0.0058	4.77	0.0304	12.3	0.0007	1.7	0.1944
Cultivar × year			1.2	0.2802	3.49	<0.0001	6.6	<0.0001	1.59	0.0762	1.1	0.3457	0.67	0.8224
Denominator d.f.	124		172		171		104		171		104		167	

### Genotypic variation in deep rooting

**Fig. 4.**
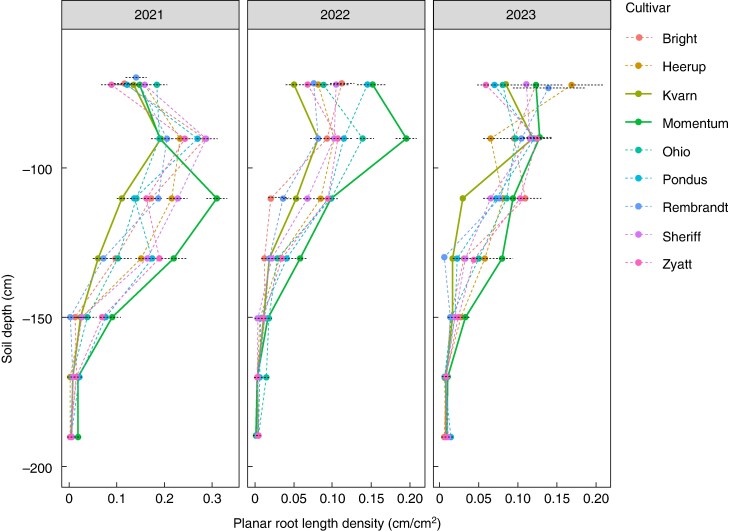
Root profiles for eight cultivars of winter wheat in June in the RadiMax facility. Planar root length density (pRLD) indicates mean root length in cm cm^2^ of image (Changdar *et al*., 2023). The mean pRLD is plotted for each 30-cm interval of soil depth; error bars represent the standard error of all root measurements within each 30-cm depth interval. Contrasting cultivars Kvarn and Momentum have solid lines.

Deep roots were visible below 1.5 m in all three years in June, with the most deep roots in 2021 and the least in 2022 (Table 2; Fig. 4). Root profiles differed between cultivars, with variation between years; however, some cultivars had consistently more or fewer roots. These cultivars, replicated at least six times in all experimental years, are shown in Fig. 4 (additional cultivars replicated four or more times are included in the rest of the analysis). Cultivars Kvarn (highlighted), Bright and Rembrandt tended to have fewer roots overall; Momentum (highlighted), Heerup and KWS Zyatt had more roots. However, there is variation between years and along the soil profile, without clear consistency. Overall, Kvarn and Momentum appear to be the cultivars with the most contrasting root systems.

Estimated genotype mean values of DeepRoot_40_ for the three years were within the range of 95–120 cm, with a 25-cm difference between Momentum, the cultivar with the deepest rooting, and Kvarn, which had the least deep roots (Table 5). Momentum had the lowest grain δ^13^C and Ohio had the highest δ^13^C. The highest δ^15^N was in Momentum and lowest δ^15^N and δ^2^H were in Kvarn. Pondus and Bright had the highest δ^2^H.

**Table 5.** Cultivar differences in DeepRoot_40_, δ^13^C, δ^15^N and δ^2^H. Reported values are estimated means from a generalized least squares model, including only cultivars that were replicated in all three experimental years (*n* = 9). Different letters indicate that they are significantly different (*P* < 0.05).

Cultivar	DeepRoot_40_ (cm)			δ^13^C, grain			δ^15^N, grain			δ^2^H, grain		
	Mean	s.e.		Mean	s.e.		Mean	Se		Mean	s.e.	
Bright	101.3	5.3	bc	−25.48	0.12	ab	19.0	5.0	ab	−50.10	2.63	a
Heerup	109.9	5.3	ab	−25.55	0.12	ab	12.8	3.4	ab	−56.62	2.34	ab
Kvarn	95.1	4.8	c	−25.56	0.11	ab	8.3	1.9	b	−59.55	1.87	b
Momentum	120.4	5.2	a	−26.24	0.12	d	25.1	6.7	a	−53.01	2.54	ab
Ohio	113.7	5.2	ab	−25.43	0.12	a	19.3	5.0	ab	−56.18	2.33	ab
Pondus	107.5	5.2	abc	−25.51	0.12	ab	17.9	4.6	ab	−50.10	2.66	a
Rembrandt	102.4	5.8	bc	−25.81	0.13	bc	11.8	3.6	ab	−58.74	2.45	ab
Sheriff	107.9	5.2	abc	−26.07	0.11	cd	17.5	4.5	ab	−53.47	2.37	ab
Zyatt	114.2	5.3	ab	−26.17	0.12	cd	13.3	3.5	ab	−56.18	2.34	ab

**Fig. 5.**
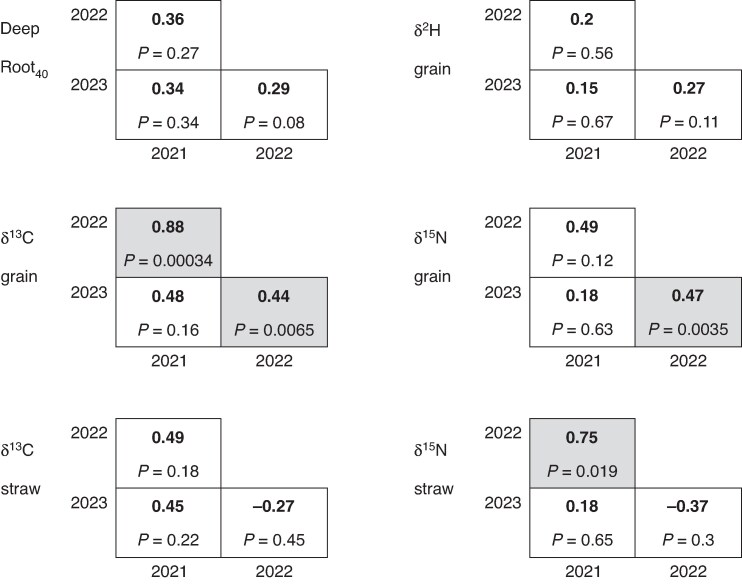
Correlations of cultivars across years: DeepRoot_40_ in June; grain δ^2^H; grain δ^13^C; grain δ^15^N; straw δ^13^C; and straw δ^15^N in 2021–23 in the RadiMax facility. Correlation coefficients (*R* values (in bold)) and corresponding *P*-values are shown. Those with *P*-values <0.05 are highlighted.

DeepRoot_40_ showed a positive correlation of genotype means between years, although these were not statistically significant (Fig. 5). Correlations of grain δ^13^C were strongly positive and statistically significant between 2021 and 2022, and 2022 and 2023. Correlations of δ^15^N were positive for all years, and statistically significant between 2022 and 2023. Correlations for δ^2^H were weakly positive and not statistically significant between years. A few of the year-to-year correlations (4 out of 18) were significant, but almost all others were also positive. The only negative but insignificant correlations were related to correlations in straw values between the very wet 2022 and the very dry 2023 season.

### Roots and water stress

**Fig. 6.**
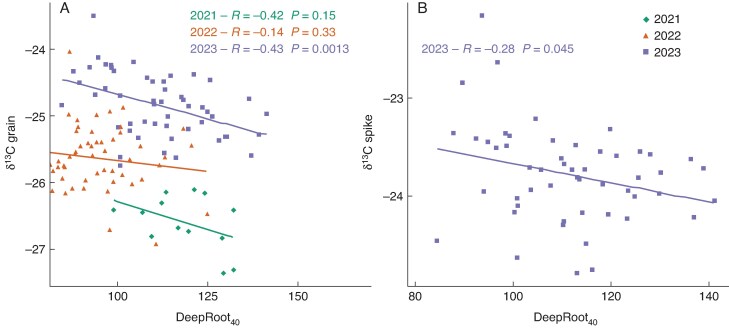
Correlations between (A) grain δ13C and DeepRoot40 by year in RadiMax winter wheat, and (B) δ13C in spike at anthesis and DeepRoot40 in 2023, and in spike at anthesis and in grain in 2023. Pearson correlation coefficients (*R*-values) and corresponding *P*-values are shown.

There was a negative correlation between DeepRoot_40_ and δ^13^C in all years, which was statistically significant in 2023 (*P* = 0.0013). Grain δ^13^C values were higher and correlated negatively with deep rooting in 2023, which was the driest year; in less dry years, grain δ^13^C values were lower and the correlation was still negative but not significant (Fig. 6A). In 2023, δ^13^C in the grain at maturity and in the spike at anthesis showed a strong positive correlation; DeepRoot_40_ was negatively correlated with δ^13^C measured in the spike at anthesis (*P* = 0.045) (Fig. 6B). DeepRoot_40_ correlations with δ^13^C in straw were not statistically significant in any year: in 2021, *R* = −0.069, *P* = 0.86; in 2022 *R* = −0.42, *P* = 0.23; in 2023, *R* = −0.42, *P* = 0.23. Grain yield was not significantly correlated with δ^13^C or with DeepRoot_40_ except in 2022, when DeepRoot_40_ correlated strongly with grain yield (*R* = 0.9, *P* = 0.00042).

### Roots and deep resource use

**Fig. 7.**
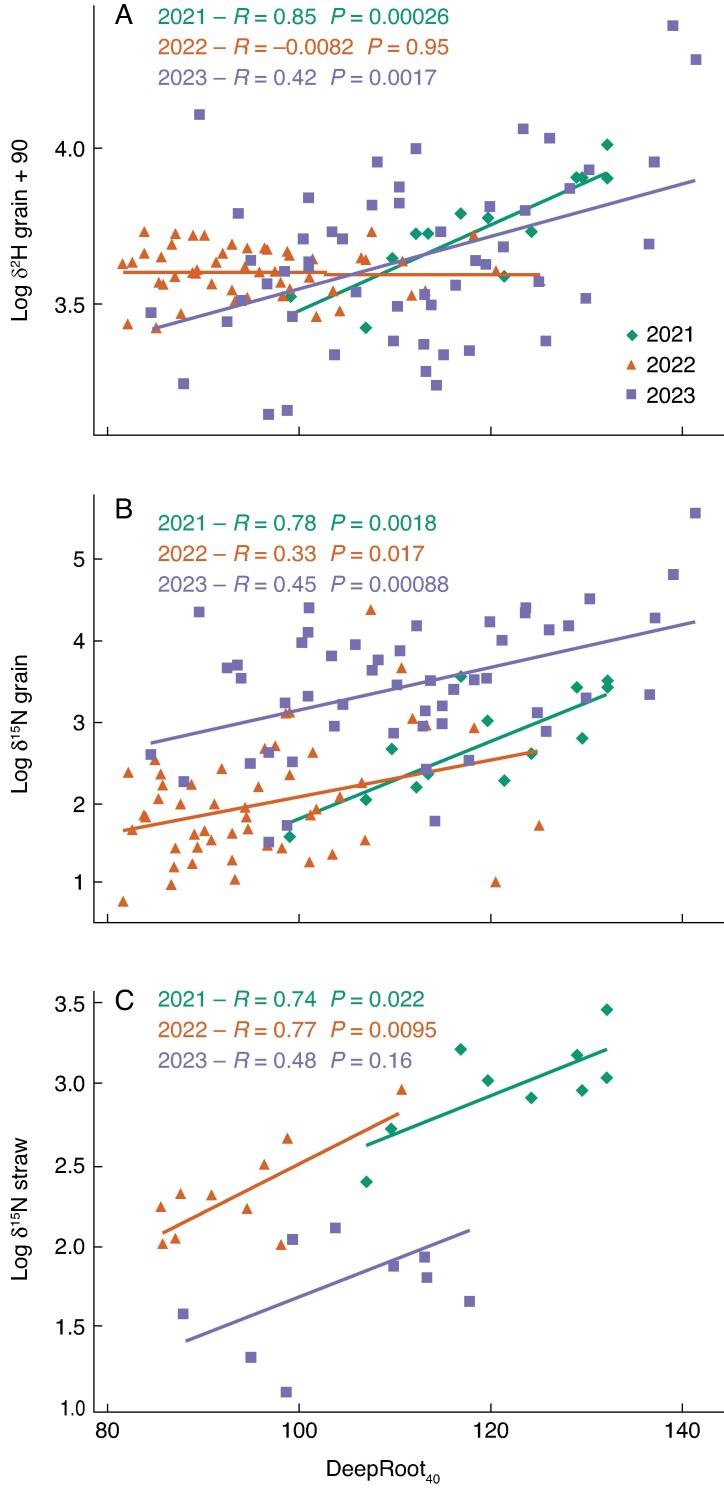
Correlations of DeepRoot_40_ in June with grain log δ^2^H and log δ^15^N and straw δ^15^N values, in 2021–23 RadiMax winter wheat. Pearson correlation coefficients (*R*-values) and corresponding *P*-values are shown.

DeepRoot_40_ was strongly positively correlated with both grain δ^2^H and grain δ^15^N in 2021 and 2023; in 2022 there was a weakly positive correlation between DeepRoot_40_ and δ^15^N (*P* = 0.017), but no correlation between DeepRoot_40_ and δ^2^H (Fig. 7). Straw δ^15^N was strongly positively correlated with deep rooting in 2021 and 2022. The SI measure of deep rooting was more weakly correlated with all three isotopes than the DeepRoot_40_ trait (Supplementary Data Figs S1–S3). Soil depth intervals from 1–1.64 m were most strongly correlated with isotopic measurements in 2021, from 1.16–1.48 m in 2022, and 1–1.70 m in 2023 (Supplementary Data Figs S1–S3). Both SI and depth intervals were strongly correlated with DeepRoot_40_ in all years.

**Fig. 8.**
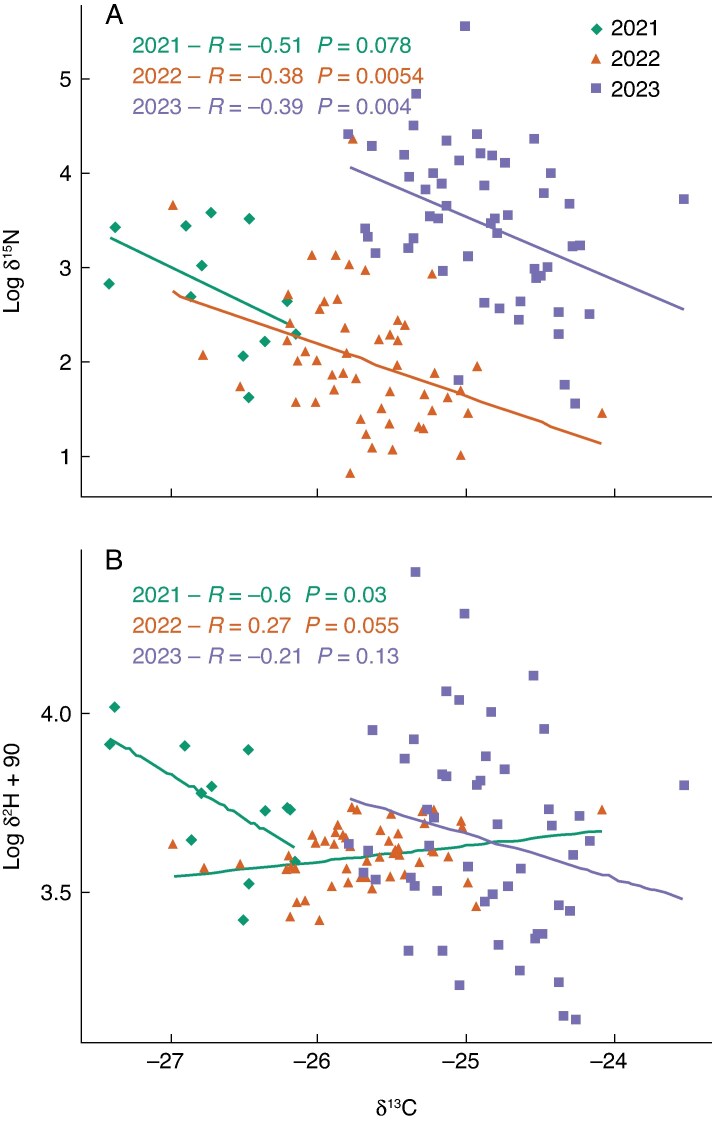
Correlations between grain δ^13^C and log δ^2^H + 90 and log δ^15^N, in 2021–23 in RadiMax winter wheat. Pearson correlation coefficients (*R*-values) and corresponding *P*-values are shown.

There was a negative correlation of δ^13^C with δ^15^N in all three years (Fig. 8) and with δ^2^H in 2021 and 2023, although not statistically significant in 2023. In 2022 there was a generally low δ^2^H level, and a non-significant positive correlation between δ^13^C and δ^2^H.

**Fig. 9.**
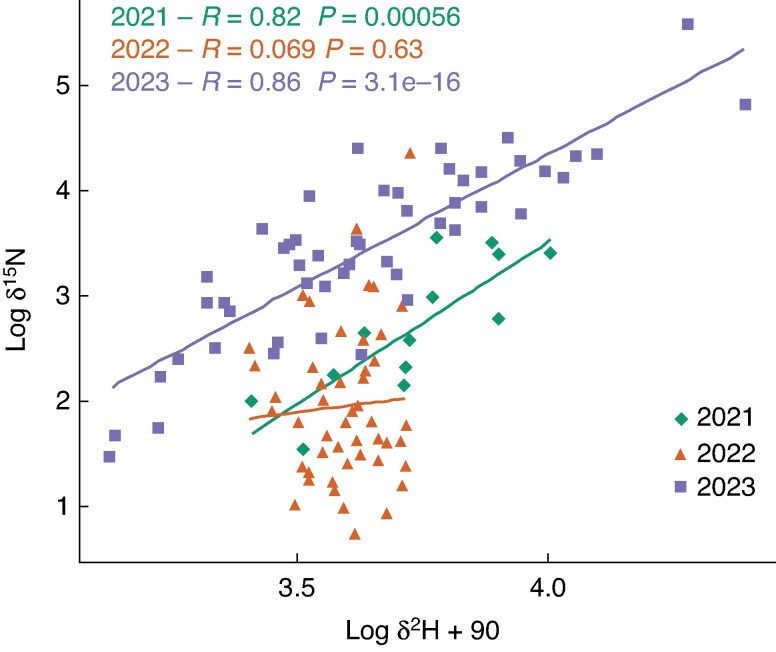
Correlations between log δ^2^H + 90 and log δ^15^N, in 2021–2023 in RadiMax winter wheat. Pearson correlation coefficients (*R*-values) and corresponding *P*-values are shown.

Enrichment of the two isotopic tracers in the grain showed a strong positive correlation in 2021 and 2023; in 2022 it was weakly positive (Fig. 9).

## DISCUSSION

### Are there genotypic differences in deep rooting and can we phenotype for them?

We have observed that certain cultivars have deeper roots and, over multiple years, we can differentiate between the cultivars with the deepest and shallowest roots, as measured by the DeepRoot_40_ trait. The correlations of DeepRoot_40_ between years were positive but not significant, but the correlation between 2022 and 2023 data, when a larger number of cultivars were replicated, had a *P*-value of 0.08. Considering the variability in soil conditions in the semi-field facility, as well as the innate uncertainty of detecting roots at deeper soil depths where they are sparse, and observation variability is high, these correlations suggest that deep rooting does vary significantly among cultivars, and clear differences can be seen in the rooting profiles between contrasting cultivars Momentum and Kvarn across the three years. The root results are backed up by the isotopic results, which while more indirect than visible roots, show root activity. The ^15^N and especially ^13^C results show stronger correlations between the years.

The extent of the difference in deep rooting is also important to consider. Over 3 years, the estimated mean DeepRoot_40_ was 25 cm higher in Momentum, the deepest-rooting cultivar, compared with Kvarn, the shallowest (Table 5). DeepRoot_40_ measures the soil depth threshold below which there is a substantial quantity (40 cm) of root length; in Momentum this threshold was on average 25 cm deeper in the soil than in Kvarn. The standard error of DeepRoot_40_ for the relatively similar modern Danish genotypes in the present study ranged between 1 and 1.9 cm, while in a previous study of winter wheat in RadiMax with a more diverse set of European and North American varieties, the standard error was 4–4.9 cm, under normal N fertilization (Odone *et al*., 2023). This suggests that more genetically diverse cultivars would have greater variation in their rooting patterns. In this study, the estimated DeepRoot40 showed a 25 cm range, compared with 37–60 cm range in RadiMax experiments in 2019–20 (Odone *et al*., 2023) and maximum rooting depth differences of 17–39 cm in Australia (Botwright Acuña *et al*., 2007) and 30 cm of difference in maximum rooting depth between winter wheat genotypes in Denmark (Rasmussen *et al*., 2015).

### Do deep roots contribute to deep soil water and N uptake?

Our results show that an increase in deep rooting can significantly enhance deep resource use, particularly water uptake. In the topsoil, where roots are relatively dense, additional root growth has limited impact, as it will mainly increase the competition between roots (Zhang *et al*., 2020). However, in the deepest soil layers where resource uptake efficiency is limited, even small increases in root exploration can increase N and water uptake, particularly during later stages of development (Thorup-Kristensen *et al*., 2020). As the limited root length density and short duration of root activity in the deepest part of the root zone limit total uptake, even modest increases in these deep layers can significantly increase uptake of water and nitrate (Robinson, 1996). While small increases are important, the observed 25 cm is quite a substantial difference. Our results show that the genotype Momentum, which had a DeepRoot40 value 26 % higher than Kvarn, had significantly higher δ^15^N and δ^13^C was significantly lower. The genotypic difference in ^13^C between the deepest and least deep-rooting varieties was roughly half the general difference between the normal year of 2021 and the very dry year of 2023, so equivalent to half the effect of a severe drought. Similarly, the ^15^N uptake was very different among genotypes, and the ^15^N uptake of Momentum was approximately three times as high as that of Kvarn. These examples show that the observed differences in rooting depth are highly significant for root function, which is also confirmed by the correlations of DeepRoot_40_ with ^13^C suggesting significant reductions in water stress and ^15^N showing increased deep N uptake.

Correlations between DeepRoot_40_ and uptake of isotopic tracers show that deep roots contributed significantly to uptake of ^15^N and ^2^H from 1.8 m soil depth in 2021 and 2023. The high statistical significance of the correlations show that deep-rooting estimates acquired by these methods certainly explain some of the variability in deep resource uptake. The low correlation coefficients can be attributed to the small sampling areas, small root observation areas on the minirhizotrons and the single-row plots in the facility. When clearly significant relationships can be obtained in spite of these methodological limitations, the real correlations must be expected to be substantially stronger. Rasmussen *et al*. (2015) showed that there were genotypic differences in deep N uptake, with a deeper-rooting genotype (Hereford) using more deep N.

While root measurements show root development at specific time points, the tracers used here indicate total uptake over an extended time period. The ^15^N was applied at anthesis; therefore it identifies genotypic differences in post-anthesis uptake rather than total N use (Barraclough *et al*., 2014). This is important as it indicates genotypes that have potential for uptake of soil N from the deepest roots, which develop later, which may contribute to reducing N leaching losses. The two tracers, ^15^N and ^2^H, were strongly correlated except in 2022. Results from 2022 indicate some contribution of deep rooting to ^15^N uptake, but not to ^2^H. The differences in water uptake from the water sensors help to explain these otherwise atypical isotope results, where deep root growth was limited compared with the two other years, and most of the water uptake was from around 0.5 m, with little uptake from the deeper soil. Higher uptake of deep-placed N than water has been demonstrated by Chen *et al*. (2021). We tried to apply the isotopic labelling at the deepest part of the root zone; however, this varied from year to year, and although we used root imaging to indicate the root depth we did not know it precisely. In 2022 we therefore applied the isotopes below the optimal location for deep roots, while in 2021 and 2023 we were more precise. The high levels of isotope uptake in 2023 indicate that we could have injected isotopes slightly deeper in order to better differentiate between deep-rooting genotypes.

### Do deep roots reduce water stress?

Deep-rooting genotypes were less water-stressed during late development. This is evident from correlations of δ^13^C with both isotopic tracers and the data from the root images. In particular in grain, δ^13^C was lower in cultivars with deeper roots, showing that deep roots were accessing soil with residual water during late development, while deep roots and straw δ^13^C were not correlated. Additionally, in 2023, the stronger correlation between deep roots and δ^13^C at maturity in grain than in spikes at anthesis reflects that ^13^C measures the effects of water stress over a long period, and indicates that it is in grain filling that deep rooting affects water stress the most. This reflects the fact that winter wheat starts the growing season with an established deep root system (Rasmussen and Thorup-Kristensen, 2016) in a soil saturated with water after the winter season. It will take a significant precipitation deficit to deplete the available soil water pool, making significant drought stress much more likely during grain filling than earlier in the season. This dynamic may be quite different with spring-sown crops, which start the season with no established root system.

Deep soil water uptake is important for grain yield, as roots only reach the deep soil in later developmental stages where water is needed for flowering and grain filling. The differences in water relations between the years can be seen in both the soil water sensors and the isotope data, which indicate strongest water use from the deep soil layers in 2023, and also in the rooting profiles, which show that root development was deeper in 2021 and 2023. In 2022 very little water was used from the deeper soil, and root development at depth was also limited, while in 2023 there was again deeper rooting. The low precipitation in the spring and early summer of 2023 reduced grain yields significantly, compared with the previous years.

The difference in grain yield between the shallow and deep sampling areas of the facility demonstrate the effect that deep water uptake can have on yields. Deeper rooting may allow continued water uptake, delayed senescence, continued photosynthesis and higher yields (Lopes and Reynolds, 2010; Gregersen *et al*., 2013). Kirkegaard *et al*. (2007) show the importance of subsoil water on yields, with an additional estimated 62 kg ha^−1^ grain yield for every 1 mm of extra subsoil water use. Ober *et al*. (2014) showed that genotypes with more deep water uptake had less yield reduction. The final experimental year, 2023 was a dry year in Denmark, where grain δ^13^C values were higher and correlated negatively with deep rooting. The strong significance of the correlation between deep roots and δ^13^C (*P* = 0.0013) in 2023 shows that the visible roots seen in the RadiMax facility explain some of the genotypic differences in water stress tolerance. Given that the minirhizotron only accesses a small observation area of the soil, covering 0.0125 m^2^ soil observation area for every 0.25 m depth interval, the correlation coefficient of 0.43 is substantial. In less dry years, the correlation was not significant, indicating less reliance on deep water for growth. Grain yield samples in RadiMax are very small; however previous studies have shown strong correlations between grain yield and grain δ^13^C, indicating that root access to water during grain filling is a major determinant of grain yield (Hafsi, 2000).

### Is the RadiMax facility effective for deep root phenotyping?

The genotype differences and correlations with isotopic tracers demonstrate that the RadiMax facility can effectively phenotype for root differences; both visible roots and their functional traits are affected by genotype. There has been a lack of studies showing deep roots under field conditions, and especially showing root development coupled with function. Previous studies in the RadiMax facility have demonstrated genotypic variation in deep rooting (Wacker *et al*., 2022; Odone *et al*., 2023), and Wasson *et al*. (2014) found variation under field conditions using soil cores. However, until now differences in deep rooting have not been demonstrated under field conditions on such a scale, showing both visible roots and their function. The present study shows a stronger data set over multiple years, with more replicates. With the capacity for up to 600 crop rows and the capacity for tracer injection, the facility provides the possibility for broader phenotyping and a deeper understanding of the significance of variation in root traits. Additionally, the facility is designed specifically for the phenotyping of deep rooting, which allows the targeting of deep roots as a breeding trait. Some other semi-field root research facilities also allow investigation of fine root development in real soil conditions; their permanent installation avoids the destruction and interference of in-season minirhizotron installation or soil coring. These include root windows (Vetterlein *et al*., 2020) and minirhizotron facilities that allow imaging of deep roots to a certain extent (to 120 and 175 cm) (Smit *et al*., 1994; Lärm *et al*., 2023). However, there is a lack of facilities capable of supporting deep-root-specific research, which is essential for future crop development (Pierret *et al*., 2016), and many existing facilities only allow a small number of repetitions or variables. Until now it has not been possible to analyse roots of large numbers of lines using minirhizotrons and deep tracer injection; in this way, RadiMax is a uniquely valuable resource.

The RadiMax facility shows genotypic differences under specific conditions, with sandy loam soil and under local weather conditions, albeit with rain-out shelters. Therefore, it will reflect root–soil interactions specific to the soil conditions, disease burden and other conditions unique in RadiMax and the individual season. Therefore, while the implications of deeper rooting for deep resource use are likely to be applicable more generally, the specific interactions of genotype and environment may vary, and the relationship between RadiMax data and root growth at other sites needs to be tested (Odone and Thorup-Kristensen, 2025*a*). For use under general field conditions, minirhizotron tubes can be laborious to install, while the tracer injections in RadiMax are also rather unique and not adapted to use in the field. However, the strong correlation with δ^13^C, particularly in a dry year, suggests that using direct measurements of ^13^C in the field could have potential as a relatively easy proxy of deep rooting in the field. This method can also be more easily transferred to different locations. Further research is needed to develop methods that can be applied in different locations and treatments, and on a large scale, for example by assessing canopy temperature as a proxy for deep rooting under drought stress (Lopes and Reynolds, 2010; Li *et al*., 2019).

Within the RadiMax experiments, although cultivar × year interactions were not significant for DeepRoot40, they were significant for δ^13^C and grain ^15^N. While ^15^N is an applied tracer, and our exact procedures and timing may have varied slightly, δ^13^C is a measure of naturally occurring isotopic discrimination, which suggests that the role of deep roots in water uptake varied from year to year. This is also evident from the very different use of subsoil water between the three years, particularly in 2022 with limited deep rooting and deep soil water use. Under different field conditions, the value of deep rooting will interact with the soil and weather, which determine whether deep water will be available and needed.

### Is the DeepRoot_40_ trait the best measure of deep roots?

Many attempts have been made to define deep rooting (Maeght *et al*., 2013), and the definition is likely to vary depending on conditions, as different depths are likely to be relevant for deep soil uptake of water and nutrients. When comparing root length across the soil depth intervals, we found that root data from the deeper layers showed the best correlations with isotopic tracer uptake (Supplementary Data Figs S1–S3), but still DeepRoot_40_ came out as the trait with the best general correlations across years and isotopes. Previous semi-field studies have shown that using a cumulative measurement such as DeepRoot_40_ is an effective measure of deep roots important for deep soil N and water uptake (Wacker *et al*., 2022; Odone *et al*., 2023). This trait works well with minirhizotron images, which show sections of roots rather than intact root systems; it includes more images than a single maximum rooting depth, and thereby reduces errors, and incorporates root density as well as root length, which is important in deep soil, where there are few roots and little competition. It should be noted that while DeepRoot_40_ is useful for comparing deep rooting, it is not an estimate of the depth of the root zone, which is deeper, as also shown by the ^15^N and ^2^H uptake. As DeepRoot_40_ underestimates the actual rooting depth, it may also underestimate the differences in total rooting depth among cultivars. The correlations between DeepRoot_40_ and labelled isotopes indicate that DeepRoot_40_ is a good measure of deep rooting, and DeepRoot_40_ in June, when deep root growth was optimized, was the best correlated with isotope uptake among the other root measurements we compared.

### Conclusions and implications

The results of this study indicate that there are substantial differences in deep rooting that we can phenotype for even under field conditions. The methods used here are unique in simultaneously measuring the location and function of deep roots in the soil, based on the design of the root phenotyping facility used for the study. The methods should be developed to allow similar measurements to be made under more common field conditions, to allow active breeding for deep rooting, contributing to crop resilience.

The results show that deep roots contribute significantly to water and N uptake, the contribution to water uptake particularly important under drier conditions. The lower δ^13^C values in deeper rooting genotypes, particularly in the drier year, show that deep roots are important for reducing water stress, and that deep rooting should be pursued as a desirable trait for ensuring yield stability in the face of climate change. Additionally, the strongly increased uptake of deep N indicates important roles for deep roots in reducing N leaching loss.

Although historically roots have not been included in breeding programmes, the results of this study indicate the importance of ensuring that deep roots in particular should be studied. Genotypic differences in deep rooting can be found, and these differences have a significant impact on function. Further development of methods such as these is needed so that roots (specifically deep roots) can be included in breeding and in crop research generally.

## Supplementary Material

mcaf160_Supplementary_Data

## SUPPLEMENTARY DATA

Supplementary data are available at *Annals of Botany*  online and consist of the following: Figure S1: correlation matrix of variables in 2021. Figure S2: correlation matrix of variables in 2022. Figure S3: correlation matrix of variables in 2023. Table S1: variety list of main cultivars tested.
