# Supplementary material for: Winter wheat phenotyping for deep root growth and function, reduced water stress and increased uptake of deep N and water
Source: Ann Bot. 2025 Aug 1;136(5-6):1101–14. doi: 10.1093/aob/mcaf160 (PMC12682833; doi:10.1093/aob/mcaf160)
Supplement: mcaf160_Supplementary_Data [file mcaf160_supplementary_data.docx]

# Supplementary material

Figure S1. Correlation matrix of variables in 2021. SI is sigmoid inflection (see Changdar et al., 2023), RL indicates root length at soil depth intervals indicated (in cm).


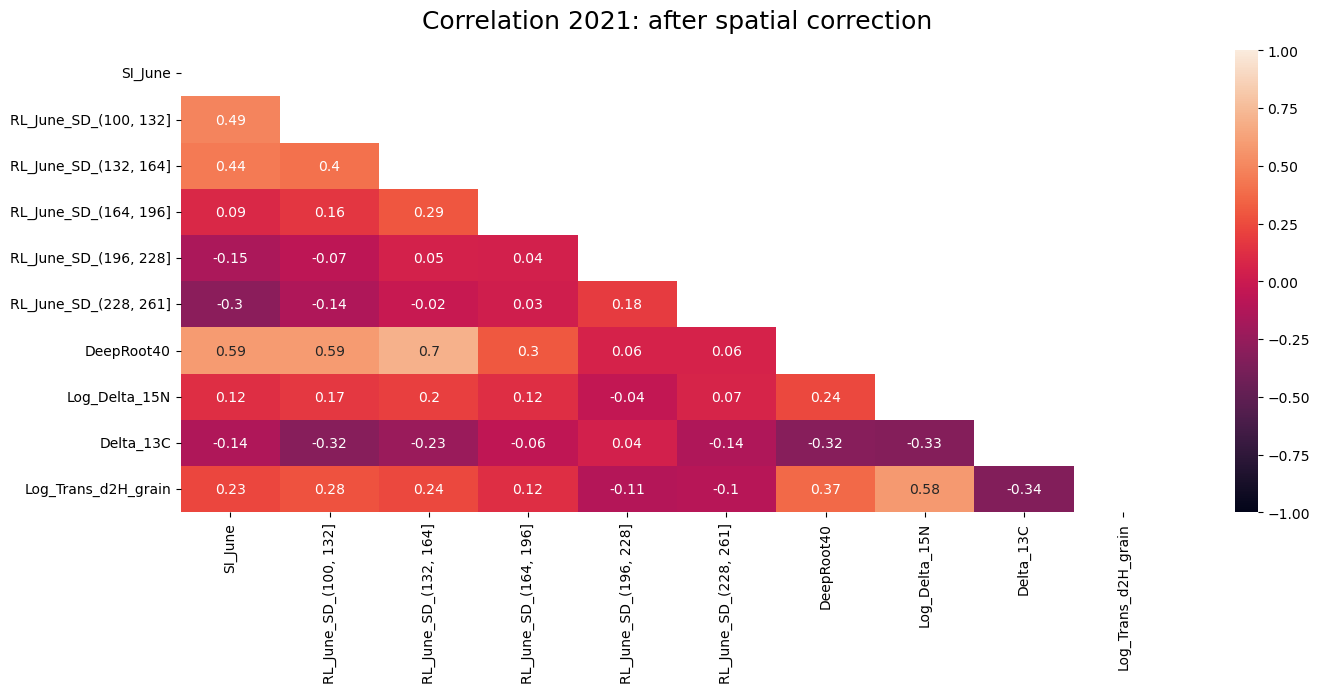


Figure S2. Correlation matrix of variables in 2022. SI is sigmoid inflection (see Changdar et al., 2023), RL indicates root length at soil depth intervals indicated (in cm).


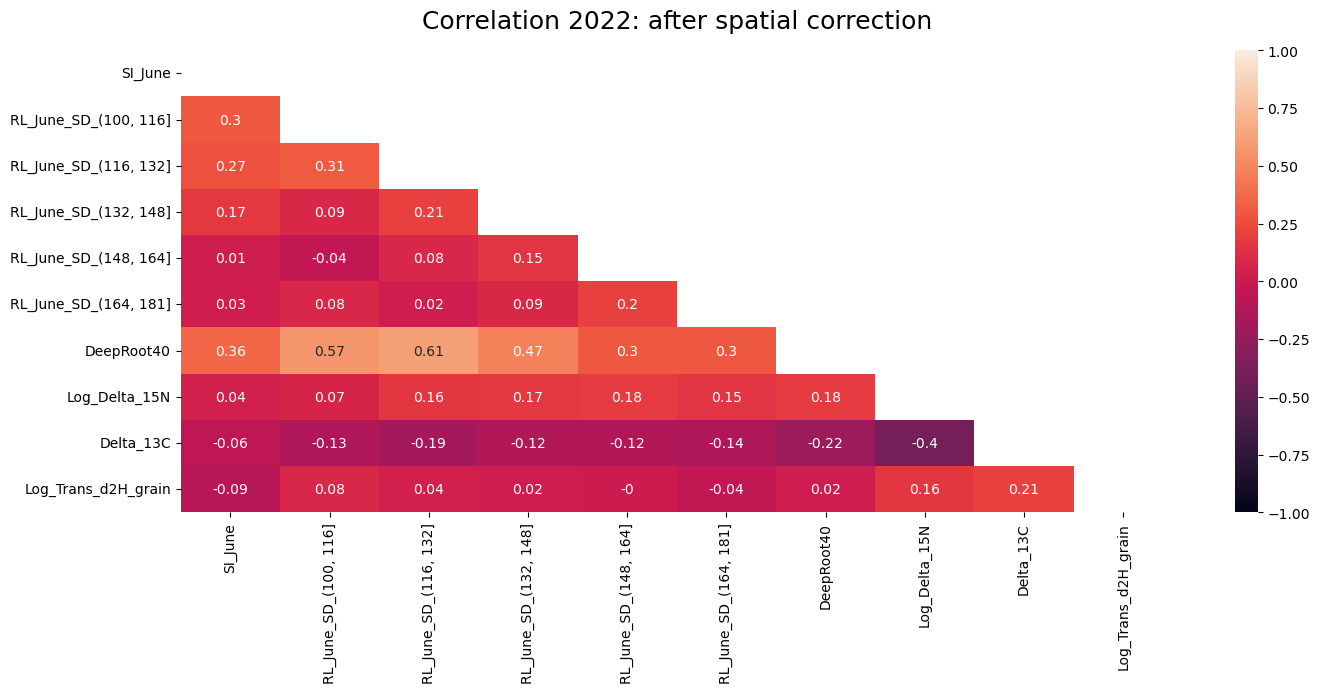


Figure S3. Correlation matrix of variables in 2023. SI is sigmoid inflection (see Changdar et al., 2023), RL indicates root length at soil depth intervals indicated (in cm).


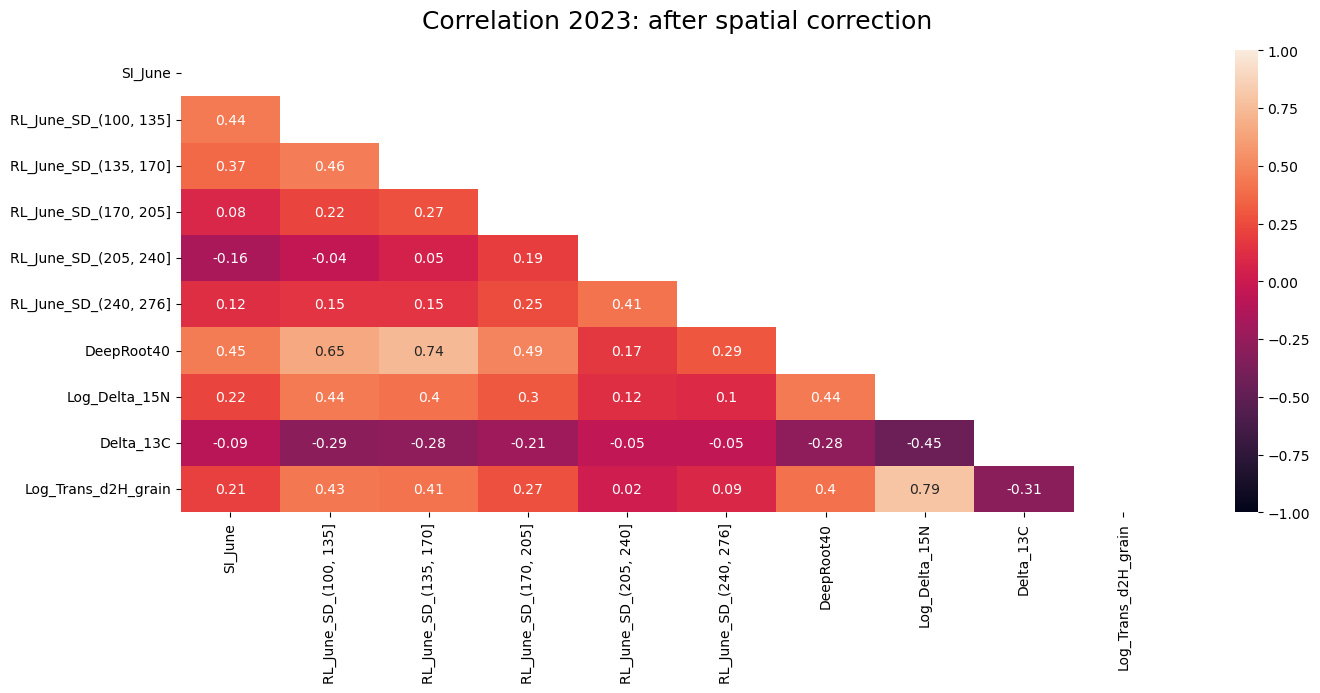


Table S1. Variety list of main cultivars tested, including those repeated at least 6 times in all years.

| Variety | # Replications | | | Breeder | Year Variety Registered | Year Reg. Ended |
| --- | --- | --- | --- | --- | --- | --- |
|  | 2021 | 2022 | 2023 |  |  |  |
| Bright | 6 | 10 | 10 | Sejet Planteforædling | 2019 |  |
| Heerup | 6 | 10 | 10 | Sejet Planteforædling | 2020 |  |
| Kvarn | 18 | 10 | 10 | Nordic Seed | 2017 | 2023 |
| Momentum | 6 | 10 | 10 | Nordic Seed | 2019 |  |
| Ohio | 6 | 10 | 10 | WvB Eckendorf | 2014 | 2024 |
| Pondus | 6 | 10 | 10 | Nordic Seed | 2020 |  |
| Rembrandt | 6 | 10 | 6 | Sejet Planteforædling | 2020 |  |
| Sheriff | 6 | 10 | 10 | Sejet Planteforædling | 2015 | 2023 |
| Zyatt | 6 | 10 | 10 | KWS | 2018 | 2024 |

Additionally, the following cultivars were tested with 4 repetitions within some years, as well as breeder lines.

| Allison | Kaldi | Lini | Tonnage |
| --- | --- | --- | --- |
| Bolinder | Kalmar | Malunas | Torp |
| Canon | Knut | Marly | Totem |
| Champion | Kvium | Mascula | Valhal |
| Faxe | KWS Extase | Pistoria | Voltage |
| Gedser | KWS Magic | Revolver |  |
| Hyvega | LG Initial | RGT Stokes |  |
| Informer | LG Skyscraper | Skagen |  |
